# Health Tract, No. 278

**Published:** 1867-03

**Authors:** 


HEALTH TRACT. No. 278.

### HEALTH HODGE PODGE.

INTERMARRIAGE.— Official documents show that seventeen families who married cousins had ninety-five children, forty-four of whom were idiots; twelve others were scrofulous or puny; another was a dwarf; another was deaf; so that more than one-half were deformed. Surely they can not be wise who in marrying cousins deliberately run such fearful risks. The very sight of a deformed child is a living torture to any mother's heart, and only the grave of one of them can end it.

RECREATION AND RENOVATION.— The body is recruited by a change in the form of its exercise; the mind is renovated by sleep, by profound rest; hence the best way of reinvigorating the whole man whether of the laborer or the literateur, is not to go to the springs or some country house and lounge, and loiter, and eat, and dose away the tardy hours, but to secure employment which will bring into requisition those muscles of the body which have, in a measure, been lying dormant, and to keep up that exercise in the open air day after day to an extent that the body shall be so fatigued that deep sleep comes within five minutes after the head has reached the pillow, that gives natural rest to the brain which, for the whole day following will thrill the whole body with the electrical influences which it distributes through it by means of the nervous system; and if this process is repeated day by day, it will not be a week before a new spring will be added to the step, a new fire will sparkle in the eye, a new energy will be infused into the mental faculties, and the whole physical man will be rejunevated, while heart and soul will respond to the general invigoration.

SLEEPING ROOMS should always face the south so as to secure all that is possible of the drying, life-giving and purifying influences of the sun's rays. In modern Rome this is perfectly understood by the citizens and just double is asked for a room, or a parlor or lodging-place which faces the sun that is asked for one of a Northern exposure into which a sun's ray never enters. With us, many a man builds a house so that the halls or passages are between the sun and their chambers.

THE SECRETS OF HEALTH are six:

First, Keep warm. Second, Eat regularly and slow. Third, Maintain regular daily bodily habits. Fourth, Take early and very light suppers. Fifth, Keep a clean skin. Sixth, Get a plenty of sleep at night.

